# Understanding the Tumor Microenvironment in Melanoma Patients with In-Transit Metastases and Its Impacts on Immune Checkpoint Immunotherapy Responses

**DOI:** 10.3390/ijms25084243

**Published:** 2024-04-11

**Authors:** Jiabao Tian, Camelia Quek

**Affiliations:** Faculty of Medicine and Health, The University of Sydney, Sydney, NSW 2006, Australia; jtia5428@uni.sydney.edu.au

**Keywords:** melanoma, in-transit metastases, immune checkpoint inhibitors, tumor microenvironment, immunotherapy response

## Abstract

Melanoma is the leading cause of global skin cancer-related death and currently ranks as the third most commonly diagnosed cancer in Australia. Melanoma patients with in-transit metastases (ITM), a type of locoregional metastasis located close to the primary tumor site, exhibit a high likelihood of further disease progression and poor survival outcomes. Immunotherapies, particularly immune checkpoint inhibitors (ICI), have demonstrated remarkable efficacy in ITM patients with reduced occurrence of further metastases and prolonged survival. The major challenge of immunotherapeutic efficacy lies in the limited understanding of melanoma and ITM biology, hindering our ability to identify patients who likely respond to ICIs effectively. In this review, we provided an overview of melanoma and ITM disease. We outlined the key ICI therapies and the critical immune features associated with therapy response or resistance. Lastly, we dissected the underlying biological components, including the cellular compositions and their communication networks within the tumor compartment, to enhance our understanding of the interactions between immunotherapy and melanoma, providing insights for future investigation and the development of drug targets and predictive biomarkers.

## 1. Overview of Melanoma In-Transit Metastases (ITM)

Melanoma is a significant global health concern, with a disproportionately high incidence and mortality rate in the Australian population [1]. Over 11,000 Australians are diagnosed with melanoma annually [2]. ITM are locoregional melanoma metastases that develop between the primary tumor and nearby lymph nodes. Most ITM lesions progress to nodal or distant metastases, which significantly reduce patient survival outcomes. This section delves into the biological origins of melanoma ITM, the melanoma staging system, and a broad spectrum of treatment options, aiding in our understanding of the initiation and progression of melanoma as well as the current therapeutic landscape.

### 1.1. Melanoma Biology and Staging

Melanoma is the deadliest form of skin cancer, which accounts for 75% of global skin cancer-related deaths each year [3]. It arises when the pigment-producing cells (melanocytes) undergo genetic mutations, usually triggered by excessive exposure to ultraviolet radiation, resulting in the neoplastic transformation of melanocytes [4]. Over time, the aberrant melanocytes proliferate uncontrollably and develop melanoma. Following the disease onset, melanoma likely metastasizes to the nearby lymph nodes and/or distant organs, often targeting the lungs, liver, brain, and bones [5,6]. Based on the locations of disease origin, melanoma is categorized into cutaneous, acral, uveal, and mucosal subtypes, of which cutaneous melanoma constitutes over 90% of the total melanoma diagnoses [7,8].

Once a patient is diagnosed with melanoma, the TNM (Tumor, Nodes, Metastasis) staging system developed by the American Joint Committee on Cancer is used to classify tumor severity, aiding in prognosis prediction and treatment rationalization. Stages I and II lesions, differentiated by Breslow thickness and ulceration presence, have not spread beyond the primary tumor; Stage III involves either locoregional or lymph node metastases; Stage IV includes distant metastases where the cancer spreads to distant organs [9]. Melanoma metastases can be classified into locoregional (satellite and ITM), nodal, and distant subtypes with varied long-term survival outcomes. The five-year survival rates for patients with solitary satellite metastases and ITM are 54.6% and 36%, respectively, whereas it drops to around 19% for those with distant metastases [10,11,12,13].

### 1.2. ITM of Melanoma

ITM is a unique phenomenon found in melanoma patients. It manifests as the entrapment of tumor cells within the dermal or subdermal lymphatic channels, spreading more than 2 cm away from the primary tumor but not reaching the regional lymph nodes [14,15]. Despite several studies proposing explanations for its underlying mechanisms, the precise factors that drive ITM development remain elusive [16,17,18].

ITM develops in 4–10% of patients with cutaneous melanoma either as a solitary disease (Stage IIIB) or in conjunction with the nearby lymph node involvement (Stage IIIC) [11]. ITM at Stage IIIB results in 47% 5-year survival, compared to 19% of those at Stage IIIC [9,19,20]. Most patients with ITM rapidly progress to nodal or distant metastases, whereas some may experience multiple ITM recurrences and develop disease progression much later or never [21,22]. Factors that are strongly correlated with ITM occurrence include aging (>50 years), specific anatomic locations of the primary tumor (on the head, neck, or lower extremity), and the presence of ulceration alongside Breslow thickness [23].

### 1.3. Treatments for Melanoma and ITM

The recent decade has witnessed a revolution in melanoma treatments, with the approval of a wealth of new drugs and treatment regimens. Surgical-wide excision is the first-line therapy for primary melanoma, which results in a 90% cure rate [24]. Therapeutic lymph node dissection (TLND) is recommended for local or regional metastases to reduce the likelihood of recurrence. However, the efficacy of TLND remains controversial as no significant difference has been observed in patients who have undergone TLND compared to those who have not [25]. In the meantime, perioperative drug therapies are employed either before (in neoadjuvant settings) or after (in adjuvant settings) TLND to further prevent disease recurrence or metastasis. Inhibitors of programmed cell death protein 1 (PD-1) or BRAF-targeted therapies represent the mainstay adjuvant treatments that lead to durable disease control with reduced recurrence rates and improved distant metastasis-free survivals [8]. The application of anti-PD-1 inhibitors in neoadjuvant settings is presumed to reinvigorate the lymphocytic anti-tumor activities more effectively in the presence of a larger tumor load and greater immune heterogeneity. Though currently under investigation, the neoadjuvant strategy has demonstrated promising results, as evidenced by the achievements of 95% (4-year) and 90% (2-year) overall survival (OS) in recent clinical trials [26,27]. Additionally, the anti-PD-1 checkpoint inhibitor pembrolizumab has demonstrated a 72% 2-year event-free survival rate in neoadjuvant settings, compared to 49% in adjuvant settings [28].

A variety of therapeutic strategies for patients with ITM are currently available [29]. Surgical resection of the local tumor mass is the mainstay treatment, followed by regional or systemic therapies if the cancerous region is not completely removed [21]. For unresectable ITM with bulky tumors, chemotherapies, including hyperthermic isolated limb perfusion and isolated limb infusion, are standard practices, whereas those accompanied by distant metastases are often treated systemically alongside intralesional therapies [30,31]. Systemic immunotherapies are showing remarkable results in treating unresectable ITM accompanied by other metastases. Immunotherapies targeting the checkpoint molecules expressed on immune or tumor cells, including cytotoxic T-lymphocyte antigen 4 (CTLA-4), PD-1, and programmed death-ligand 1 (PD-L1), have demonstrated outstanding clinical efficacies in recent studies and clinical trials, laying the groundwork for the development of standard ITM treatments [15,19,32,33,34].

## 2. Immunotherapy: Immune Checkpoint Inhibitors (ICIs)

Immunotherapies, particularly ICIs that target immunomodulatory receptors CTLA-4 and PD-1, or the ligand PD-L1, have transformed the landscape of melanoma treatments [35]. However, more than half of patients with advanced melanoma lack durable objective responses to ICI therapies [36,37,38]. Therefore, improvements in our understanding of tumor biology and its interaction with ICI therapies are urgently required. Here, we have outlined the common ICIs used in melanoma treatments with the underlying biological mechanisms, evidenced by the up-to-date clinical trials. We have also discussed the diverse range of cellular and molecular factors that are strongly associated with therapy response or resistance, aiding in the future development of drug targets or biomarkers.

### 2.1. Types of ICIs

**Figure 1:**
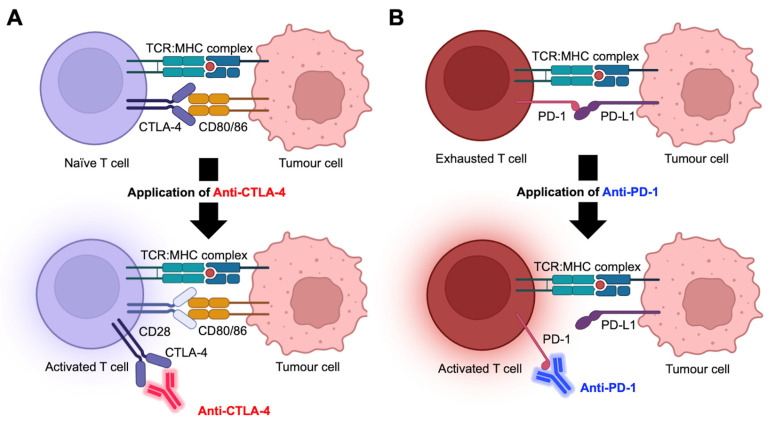
Mechanistic actions of anti-CTLA-4 and anti-PD-1 checkpoint inhibitors. (**A**) Interaction of CTLA-4 with the CD80/86 receptor prevents naïve T cells from differentiating into an activated state. Anti-CTLA-4 facilitates CD28 and CD80/86 interactions, allowing naïve T cells to activate and mature. (**B**) PD-1 binds to PD-L1 to turn T cells into an exhausted state where their anti-tumor effects are lost. Anti-PD-1 abrogates PD-1/PD-L1 interaction, thus reinvigorating T cell cytotoxic capacity. Abbreviations: TCR—T cell receptor; MHC—major histocompatibility complex; CTLA-4—cytotoxic T-lymphocyte antigen 4; PD-1—programmed cell death protein 1; PD-L1—programmed death-ligand 1. CD4+ T cells, CD8+ T cells, and tumor cells are represented as purple, crimson, and pink, respectively. Monoclonal antibodies targeting CTLA-4 and PD-1 are colored in red and blue respectively. Figures are adapted from [36], and modified using BioRender.

ICIs target the dysfunctional immune system and stimulate the anti-tumor activities of lymphocytes, particularly the CD4+ and CD8+ T cells, to suppress tumor growth. Nearly 50% of melanoma patients manifest tumor regression and durable cancer control when treated with ICI, compared to 10% historically [39]. The most intensively studied checkpoint inhibitors target CTLA-4 and PD-1 [40,41]. CTLA-4 presents on naïve CD4+ T cells, where it demonstrates a strong binding affinity for CD80/86 ligands expressed by antigen-presenting cells. This interaction dampens T cell priming, leading to the inhibited activation and proliferation of CD4+ T cells. CTLA-4 inhibitors enable CD80/86 to engage with CD28, thus promoting co-stimulation of CD4+ T cells and restoring their differentiative and proliferative capacities (Figure 1A). Likewise, PD-L1 expressed by tumor cells typically binds to PD-1 on CD8+ T cells to induce T cell exhaustion, which reduces its responsiveness to tumor antigens. Anti-PD-1 disrupts the PD-1/PD-L1 interactions and reinstates the cytotoxic functions and anti-tumor activities of CD8+ T cells (Figure 1B).

In recent decades, a wealth of clinical trials on ICI therapies have elicited durable responses and demonstrated robust clinical efficacies in melanoma patients [42,43,44,45,46,47]. In 2011, ipilimumab (anti-CTLA-4) was approved by the US Food and Drug Administration (FDA) to be the first checkpoint inhibitor applicable for melanoma treatment. Anti-CTLA-4 significantly improved patient survival outcomes and has been cross-studied for treating different cancers [48,49,50]. Later, anti-PD-1 inhibitors nivolumab and pembrolizumab were introduced, outperforming anti-CTLA-4 and resulting in a nearly three-fold higher response rate and a doubled progression-free survival (PFS) [38]. Since anti-CTLA-4 and anti-PD-1 employ distinct biological pathways to restimulate lymphocytic functions, various therapeutic strategies have combined them to extract synergistic anti-tumor effects, leading to superior clinical outcomes compared to monotherapies [37,51].

In addition to the well-studied anti-CTLA4, anti-PD-1, and anti-PD-L1 approaches, the emergence of other molecules has expanded the treatment landscape and helped overcome therapy resistance. Lymphocyte-activation gene 3 (LAG-3), a co-inhibitory receptor that highly expresses on various immune cells, binds to MHC (major histocompatibility complex) class II molecules expressed by tumor cells to negatively regulate the anti-tumor immunity [52]. In 2022, the efficacy of anti-LAG-3 combined with anti-PD-1 was first evaluated in patients with advanced melanoma, demonstrating a nearly 48% 12-month PFS compared to 36% in those treated with anti-PD-1 alone [53]. Later this year, the combined use of relatlimab (anti-LAG-3) and nivolumab (anti-PD-1) was approved by the FDA for treating unresectable or metastatic melanoma. Such dual inhibition expands the spectrum of patients suitable for ICI therapies, while the novel drug target discovered offers a new option to overcome immunotherapy-induced resistance. Other inhibitory molecules such as TIGIT (T cell immunoreceptor with Ig and ITIM domains) and T cell immunoglobulin and mucin domain-containing protein 3 (TIM-3) have also been identified with upregulated expressions in melanoma, thereby suppressing immune cell proliferation and functions [54,55]. Though yet to be approved for cancer treatment, their effects are undergoing intensive investigation in clinical trials in the context of various solid tumors and leukemia [56,57].

### 2.2. Biological Factors Associated with Response to Immunotherapy

Cellular and molecular markers that facilitate pre-treatment patient stratification are critical to enhancing treatment efficacy whilst helping spare patients from unnecessary therapy-induced toxicity. A plethora of biological markers, including immune cell infiltrates, molecular factors, and checkpoint expressions, have been identified within the tumor microenvironment (TME). Their functions on tumor development and correlation with immunotherapy response or resistance are detailed in Table 1.

Tumor-infiltrating lymphocytes (TILs), such as CD8+ and CD4+ T cells, have been associated with better response to immunotherapy. CD8+ T cells have been suggested as the most abundant lymphocyte found within melanoma TME, mainly exerting cytotoxic effects to eliminate cancer cells. The increasing abundance of tumor-infiltrated CD8+ T cells has been long recognized as a strong predictor of improved ICI therapy response and prolonged PFS in melanoma [62,68,69,70,71]. Meanwhile, the anti-tumor response of CD8+ T cells is largely enhanced by CD4+ T cells. A recent study identified that interleukin (IL)-21 produced by CD4+ T cells critically promotes CX3CR1+CD8+ T cell formation [72]. Moreover, tumor-infiltrating type I T helper (Th1)-like CD4+ T cells are able to elicit cytotoxicity against B16 melanoma, indicating the potential of CD4+ T cells to directly target and eliminate tumor cells [73]. In addition, single-cell profiling identified specific CD4+ T cell subsets that are enriched in melanoma patients who respond to ICI therapies as compared to those who did not [51,74].

Molecular factors such as interferon-γ (IFN-γ) expression and tumor mutational burden (TMB) also possess predictive power for immunotherapy response. A gene expression profile with 10 IFN-γ-related genes was capable of differentiating patients who responded to anti-PD-1 therapy from those who developed resistance [63]. Moreover, tumors with high TMB contain increasing neoantigen load, enhancing their likelihood of being recognized and targeted by immune cells. High TMB thus serves as another predictor for favorable immunotherapy response [75]. The predictive power of cytokines and the tumor antigen load indicates the important role of pre-existing immunity in enhancing immunotherapeutic efficacy.

CTLA-4, PD-1, and PD-L1 checkpoints are the main targets of ICI therapies, thus forming strong correlations with therapy response. Melanoma samples exhibiting more than 20% of CTLA-4^hi^PD-1^hi^ cytotoxic T lymphocytes (CTL) favorably respond to ICI therapies compared to those with less than 20% CTLs [62]. Interestingly, recent studies have proposed that the methylation of the *CTLA4* promoter may serve as an effective predictor of favorable response and prognosis in ICI-treated metastatic melanoma, providing genetic information in addition to the protein layer to enhance the predictive power of checkpoint expressions [76,77]. The levels of PD-1 and PD-L1 expression have been routinely tested as response-associated markers in clinical practices [78]. However, relying solely on PD-L1 provides poor predictability of immunotherapy response in melanoma patients due to their cooperating interactions [79].

### 2.3. Biological Factors Associated with Resistance to Immunotherapy

Response to immunotherapy is not universal in melanoma patients. Those who lack response develop two forms of resistance: primary resistance, characterized by the absence of initial response to immunotherapy, and acquired resistance, where an initial response is observed but later followed by relapse or disease progression [67]. Primary resistance may be attributed to factors intrinsic to the tumor itself, such as its inability to produce or present neoantigens, or extrinsic factors derived from the TME, including absent TILs, infiltrated immunosuppressive cells, and high checkpoint expressions. The key intrinsic and extrinsic biological factors that drive primary resistance development are listed in Table 1.

Regulatory T cells (Tregs) are the all-around regulators that constantly fine-tune innate and adaptive immunity to prevent the occurrence of autoimmune diseases. However, their suppressing mechanisms may greatly impede the anti-tumor immune response in the context of cancer. The activation and proliferation of anti-tumor cells are suppressed by Tregs through either contact-dependent or contact-independent mechanisms, where the former inhibits the antigen-presenting capacity of dendritic cells (DC) while the latter secrets various inhibitory cytokines, such as IL-10, IL-35, and tumor growth factor β (TGF-β), to promote an immunosuppressive TME [80,81,82]. Therefore, high Treg expression within the TME has been strongly associated with the development of immunotherapy resistance in melanoma [83].

Likewise, other immunosuppressive cells, such as myeloid-derived suppressor cells (MDSC) and M2 macrophages, contribute to immunotherapy resistance. They release anti-inflammatory cytokines such as IL-1, IL-10, and TGF-β to create an immunosuppressive environment and suppress the anti-tumor activities of T cells or NK cells. An elevated number of monocytic MDSCs with significantly higher production of nitric oxide (NO) was observed within the TME of non-responders, suggesting the immunosuppressive capacity of MDSCs [84]. Likewise, the polarization of tumor-associated macrophages towards the M2 subtype inhibits T cell cytotoxicity, which likely induces anti-PD-1/PD-L1 resistance [85,86].

In addition, overexpression of checkpoint molecules such as LAG-3 and TIM-3 on various immune cells also promotes tumor resistance to immunotherapy. Preclinical studies have demonstrated upregulated expressions of LAG-3 or TIM-3 in immunotherapy-resistant tumors, whilst co-blockade of these checkpoint molecules augments tumor response to anti-CTLA-4 and anti-PD-1 immunotherapies [67,87]. The potential of LAG-3 to predict anti-PD-1 therapy response has been investigated, which found a strong correlation between tumor-infiltrated LAG-3 expression and therapy resistance [88]. Another study observed that among patients who have progressed following anti-PD-1 therapies, those with >1% LAG-3 expression give significantly higher objective response rates (ORR) to the combined therapy (anti-LAG-3 plus anti-PD-1) than those with <1% LAG-3 expression, suggesting the potential role of LAG-3 in assisting the selection of second-line treatments for patients who progressed from the previous immunotherapies [89].

### 2.4. Application of Immunotherapy in Melanoma ITM

The efficacy of ICI therapies, including the anti-CTLA4 (ipilimumab) and anti-PD-1 (nivolumab and pembrolizumab) monoclonal antibodies, is well established in patients with stage III/IV melanoma. However, only limited immunotherapy-related studies have focused on ITM due to the small patient cohort and little understanding of its clinical presentation and disease course. Moreover, due to our limited insights into the underlying ITM biology, the unique cellular or molecular features possessed by its microenvironment and its interactions with melanoma cells remain to be defined in melanoma. A recent retrospective study found that ICI therapy gives durable responses in ITM patients similar to those with stage IV melanoma, resulting in an ORR of 54%, 2-year PFS of 39%, and 2-year OS of 63% [19]. Another study by Storey et al. assessed the disease response in 63 patients with multiple ITM and reported an ORR of 62% [90]. In these two studies, 74% and 88% of patients received single-agent anti-PD-1 therapy, respectively, and gave quite similar response rates (58% versus 59%). Another study collected multi-centric patients with unresectable ITM and observed their response to ICI therapies with a doubled median follow-up [21]. However, patient response to anti-PD-1 therapies was significantly reduced to an ORR of around 31%, potentially attributed to the inclusion of those with signs of nodal or distant metastases. Overall, these results suggest that systemic immunotherapy is able to effectively treat melanoma ITM. Since most of these studies used ICI therapy as the first-line treatment, further effort is required to examine its therapeutic efficacy when applied in multimodal scenarios, including its combination with surgery, intralesional, and/or regional therapies.

## 3. Tumor Microenvironment of Advanced Melanoma

TME is a complex and dynamic ecosystem composed of diverse cell types, including immune and stromal cells, blood vessels, and the extracellular matrix, that tightly interact with the tumor. The heterogeneous cellular composition and their intricate spatial organizations within TME substantially impact the effectiveness of cancer control and therapy response. This section dissects the immune profile of melanoma TME and discusses the cellular interplays that occur among diverse cell populations, followed by the characterization of spatial phenotypes at the tissue level, which is further related to patient response to immunotherapy.

### 3.1. Immune Characterization of Melanoma Microenvironment

The adaptive and innate immune systems form an “army” to surveil, recognize, and attack the abnormally proliferative tumor cells during tumorigenesis and development. Here, the functions of major adaptive immune populations, including T cells and B cells, and innate immune communities, such as natural killer (NK) cells, DCs, MDSCs, and macrophages, are described and discussed, picturing the fundamental immune landscape of melanoma TME.

#### 3.1.1. Adaptive Immunity

Within the immune profile, CD8+ and CD4+ T cells are the most well-studied populations, and functions of their subtypes distinctively affect cancer growth. CD8+ T cells are the major “cancer killers” that induce tumor apoptosis through cytotoxic reactions or cytokine secretion. The cytotoxicity of CD8+ T cells is carried out by two proteins, the granzymes, which invade target cells to induce apoptosis directly, and the perforins, which punch holes in cell membranes to allow for granzyme entrance. Moreover, the cytokines, such as IFN-γ secreted by cytotoxic T cells, bind to IFN-γ receptor on tumor cells to activate apoptosis signaling pathways and inhibit cancer growth.

The CD4+ T cells play dual opposing roles in tumor progression, displaying anti- and pro-tumorigenic properties [91]. For instance, CD4+ helper T cells sustain the leukocytic anti-tumor functions through cytokine secretion (IFN-γ, TNF, and IL-2) while modifying the antigen presentation of DCs and B cells via direct CD40/CD40L interactions. However, the CD25+CTLA-4+ Tregs are primarily pro-tumorigenic as they suppress the priming and differentiation of naïve CD4+ T cells. Treg suppressive functions are mediated by different mechanisms, including the release of inhibitory cytokines (IL-10 and TGF-β), inducing apoptosis in effector T cells through the Fas and Fas ligand pathway, metabolic disruption and direct cell–cell contact inhibition between antigen-presenting cells and effector T cells [92]. The precise role of how CD4+ T cell sub-populations influence the growth of tumors and immune evasion remain to be defined in melanoma patients with ITM.

The roles of B cells and the B cell-mediated humoral response in tumor growth are usually overlooked. However, tumor-associated B cells (TAB) with multiple phenotypes can make up to one-third of the immune populations within the melanoma TME [93,94]. TABs simultaneously exhibit pro- and anti-tumorigenic properties. For instance, a recent study identified the co-existence of two TAB subtypes in melanoma: the plasmablast-like TABs, which give inflammatory anti-tumor responses, and regulatory B cells, which display immunosuppressive effects. Delving into the individual cell type, they further discovered that the plasmablast-like TABs simultaneously express stimulatory and inhibitory signatures, conferring them the ability to promote or suppress anti-tumor responses in different contexts and making it a unique population within the melanoma TME [95]. The discovery of such a seemingly contradictory biological feature indicates the subtle balance between immunity and tolerance, therefore emphasizing the importance of conducting context-dependent analysis of cellular functions.

#### 3.1.2. Innate Immunity

Another aspect that critically influences tumor development regards the wide array of innate immune populations. NK cell-mediated cytotoxicity targets and kills aberrant cells through the interaction of receptor natural killer group 2D and stress-induced MHC class I chain-related protein A and B (MICA and MICB). Proteolytic shedding of MICA and MICB caused by tumor evasion promotes tumor progression, which is usually alleviated by antibody-mediated inhibition to restore the NK-driven innate immunity [96]. Moreover, the chemokine ligand 5 secreted by NK cells is capable of recruiting conventional DCs to enhance the NK-mediated anti-tumor functions [97].

DCs are another critical member within the melanoma TME, considering its ability to activate the anti-tumor functions of CD8+ T cells. They are categorized into plasmacytoid, conventional, and monocyte-derived subtypes. Conventional type I DCs (cDC1) exhibit the superior capacity to cross-present tumor antigens to CD8+ T cells, thus inducing the strongest anti-tumor immunity [98]. However, tumor-infiltrating cDC1 has limited expression in melanoma compared to other cancer types, making it an ineffective target for certain immunotherapies such as adoptive T cell transfer [99].

Anti-tumor responses elicited by TILs are constantly counteracted by the inhibitory effects derived from immunosuppressive populations, particularly MDSCs and M2 macrophages. Immunoregulatory cytokines such as IL-4 and IL-10 polarize macrophages towards the M2 phenotype, which hinders antigen presentation and aids tissue repair and remodeling, thus promoting tumor progression. Not all immunosuppressive macrophages strictly display the M2 phenotype. Therefore, their diversity and complexity in the TME require further classifications to refine clinical decision-making.

MDSCs represent another major immunosuppressive population within the melanoma TME [100]. They consist of diverse myeloid cells that mainly inhibit the anti-tumor functions of CD8+ T cells and NK cells. Since MDSCs simultaneously employ multiple suppressive mechanisms, therapies are expected to target these mechanisms concurrently to optimize clinical outcomes. Meanwhile, the phenotypic features shared among various myeloid populations greatly reduce drug target specificity. The application of single-cell technologies, thus, becomes essential to accurately define the diverse phenotypic states and enhance the targeting accuracy of immunotherapeutic inhibitors [101].

Interestingly, some therapies target the altered tumor metabolism to restore the cellular anti-tumor responses. A classic example refers to arginine deprivation therapy for treating malignant melanoma. Arginine is a molecule that promotes NO production, while arginosuccinate synthetase, the enzyme that degrades arginine, is usually downregulated in melanoma cells. As a result, redundant arginine produces abnormally high levels of NO, which greatly impedes the anti-tumor functions of T cells and NK cells. By degrading the redundant arginine in tumor cells and restoring the anti-tumor activities within the TME, arginine deprivation therapy has become a promising treatment for malignant melanoma [102].

### 3.2. Cell–Cell Communications in Melanoma Microenvironment

Communications between aberrant cells and diverse immune communities help shape tumor immunity. At the initial stage of tumorigenesis, DCs present tumor neoantigens to prime and activate T cells, empowering the latter with anti-tumor functions to suppress tumor progression. However, tumors have evolved escape mechanisms that help them evade immune attacks. For instance, they upregulate the inhibitory receptor PD-1 on CD8+ T cells, which binds to PD-L1 on tumor cells, to suppress the anti-tumor cytotoxic effects [103]. A tug-of-war is thus created between the immunity-mediated anti-tumor responses and tumor-mediated escaping mechanisms to dictate tumor elimination or immune resistance.

Immunotherapy resistance arises when the balance tips toward the tumor-mediated escape. Key resistance-associated cell types include cancer-associated fibroblasts (CAFs), MDSCs, M2 macrophages, and Tregs. Papaccio et al. explored the reciprocal interactions between melanoma and CAFs and observed the increasing expression of the growth factors *b-FGF*, *SCF*, *VEGF*, and *MMP1* in the melanoma/CAF coculture compared to those in a CAF monoculture [104]. This finding highly suggests the paracrine interactions that occur between these two cell populations. Notably, cell–cell communications underpin the pharmacological basis of immunotherapy, with virtually all therapeutic strategies utilizing direct ligand–receptor interactions to initiate an anti-tumor immune response. ICI therapies, for instance, use monoclonal antibodies to block receptors from binding with their corresponding ligands, thus preventing T cell suppression and reinstating their anti-tumor functions.

### 3.3. Inflamed and Non-Inflamed Phenotypes of Melanoma Microenvironment

**Figure 2:**
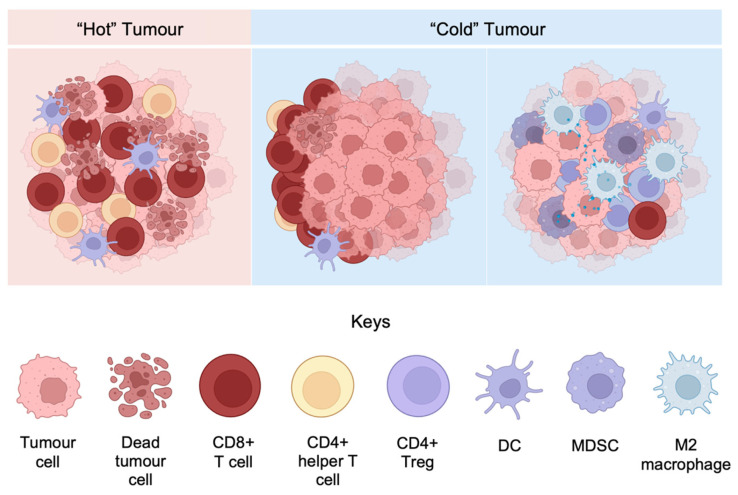
Differentiation of “hot” and “cold” tumors based on the patterns of immune cells. The “hot” tumor TME contains a high level of activated tumor-infiltrated TILs, DCs, and helper T cells (**left**). The non-inflamed, immune-excluded tumors display abundant anti-tumor lymphocytes confined to the margin (**middle**). The non-inflamed, immune-desert TME lacks TILs but contains various immunosuppressive cells (**right**). Abbreviations: CD4+ Treg—CD4+ regulatory T cell; DC—Dendritic cell; MDSC—Myeloid-derived suppressor cell. Figures are adapted from [107], and modified using BioRender.

Melanoma TMEs are generally classified into two basic immune profiles: “hot” tumors with the immune-inflamed phenotype, and “cold” tumors with the immune-excluded or the immune-desert phenotype [105]. As illustrated in Figure 2, immune-inflamed tumors display a large number of tumor-infiltrated immune cells, specifically CD8+ T cells, within the tumor parenchyma. The large quantity of proinflammatory cytokines and chemokines produced by the TILs promotes tumor control and improves survival outcomes. On the contrary, the presence of TILs in immune-excluded tumors is mostly confined to the stromal area, with a very small number penetrating to the tumor parenchyma. Immune-desert tumors are found to lack TILs in either the parenchyma or stroma. The inflamed tumors predict favorable clinical outcomes due to the high level of active TILs, which creates an immune-activating environment and enhances tumor antigen presentation. In comparison, non-inflamed TMEs exhibit poorer immunotherapy response owing to the exclusion or absence of infiltrated T cells as well as the upregulated immunosuppressive populations. Different approaches should be considered in the display of different tumor phenotypes to optimize therapeutic benefits [105]. Nevertheless, the study by Tumeh et al. shows that an increase in CD8+ T cell density both at the invasive margin or tumor parenchyma predicts favorable clinical responses for metastatic melanoma receiving anti-PD-1 inhibitors, providing inconsistent evidence against the general “hot” and “cold” tumor theory [106]. It is thus highly suggested that multiple factors be combined to generate accurate predictive outcomes.

The immunogenicity of “hot” tumors is mainly conferred by the infiltrated CD4+ and CD8+ T cells. Despite their anti-tumor functions, CD8+ T cells readily become exhausted with a loss of cytotoxicity under chronic inflammatory pressure. However, exhausted T cells can be reinvigorated by anti-PD-1 immunotherapy to restore the cytotoxic functions [108]. CD4+ T cells are broadly divided into Tregs and helper T cells, which regulate immune responses and assist immune cell activation, respectively. Tregs aid in tumoral escape from immunosurveillance by mediating the various suppressing mechanisms: (1) the IL-2 receptors on Tregs bind to the surrounding IL-2 to reduce IL-2 availability for effector T cells, thus depriving their anti-tumor activities; (2) the constitutive expression of CTLA-4 on Tregs competitively binds to CD80/86 ligands on DCs, preventing them from activating CD8+ effector T cells [109]. On the other hand, helper T cells play positive roles in activating CD8+ T cells, while they also drive antibody production by B cells to promote tumor cell elimination.

### 3.4. Melanoma Microenvironment in the Spatial Context

Many current immunotherapies work by disrupting cell–cell interactions. In parallel, the cellular spatial distributions within the TME also play a critical role in affecting tumor growth and response to immunotherapy [110]. The spatial architecture of the TME has three broad characteristics: the spatial location of non-tumor cells within the tumor compartment, the physical distances between individual cells and their nearest neighbors, and the universal spatial patterns formed by specific cell types [111].

Cellular locations within the TME are broadly categorized as either parenchymal infiltration or stromal confinement [112,113]. In tumors with high lymphocytic infiltration, tumor cells tend to be closely located with immune cells, which implicate stronger and tighter tumor–immune interactions. In comparison, lymphocytes confined to the stroma contact with only the outermost tumor cells, largely reducing the likelihood of tumor–immune interactions and resulting in minimal anti-tumor activities.

Information derived from the cellular spatial locations is insufficient to truly reflect tumor–immune interactions, which needs to be confirmed by measuring the distances between each cell and its neighboring cells. Cellular communications between one and its neighboring cells form a highly organized biological community termed the spatial neighborhood. Unlike spatial locations, neighborhoods provide enhanced clarity on the distance between cells to predict their interactions, as entities located in proximity are more likely to interact. Previously, studies have mapped out the spatial landscape of melanoma at single-cell resolution and identified 10 different spatial neighborhoods, each of which exerts distinctive immune effects [114]. For instance, the one enriched in activated PD-1+ CTLs displays anti-tumorigenic attributes, whereas those that co-occurred with PD-L1+ myeloid cells counteract anti-tumor immunity.

Patterned spatial architectures formed by specific immune cell distributions demonstrate intertumoral and interpersonal consistency and exhibit profound clinical values in predicting therapy response or survival outcomes. A classic example refers to the tertiary lymphoid organs (TLSs) that form in many solid tumors, including melanoma [115]. These TLSs are ectopic hyperplasia lymphoid structures that consist of two major parts: the T cell-enriched zone accompanied by mature DCs, and the B cell-enriched zone with follicular DCs, plasma cells, and the secreted antibodies. Such an immune cell reservoir promotes anti-tumor immunity via T cell-directed cytotoxicity and B cell-directed antibody-dependent cellular cytotoxicity. Their clinical values in promoting immunotherapy response and improving patient survival outcomes have been suggested in recent studies [60,116].

## 4. Conclusions

Melanoma consists of diverse subtypes, of which the locoregional ITM presents as a unique phenomenon and leads to further tumor progression. Immunotherapies, specifically ICIs, have demonstrated remarkable efficacy in treating melanoma and the ITM. Various cellular and molecular factors have been tightly associated with developing response or resistance to ICI therapies. The highly heterogeneous and dynamic melanoma microenvironment, evidenced by the complex cellular composition and spatial architectures, provides further insights into the intertwinement between tumor cells and the immune system. The critical influence the TME exerts on tumor growth necessitates further investigation into finely dissecting and characterizing cellular subtypes and TME profiles to help advance our knowledge in developing novel drug targets and biomarkers.

## Figures and Tables

**Table 1:** Biological factors in TME associated with immunotherapy response and resistance.

Factors	Descriptions	Association with Response/Resistance	References
**Response**			
CD8+ T cells	Releasing IFN-γ, granzyme B, and perforin to eliminate tumor cells	High intra- or peri-tumoral proportion	[58]
CD4+ helper T cells	Assisting CD8+ T cells; secretion of IFN-γ and TNFα to directly target tumor cells	High proportion in tumor	[59]
CD20+ B cells	Forming tertiary lymphoid structure within the tumor	High proportion in tumor	[60]
M1 macrophages	Tumor-associated macrophage secreting IL-1 and TNFα that harm tumor cells	High proportion in tumor	[61]
CTLA-4	Inhibiting CD4+ T cell differentiation and activation	High expression on naïve T cells	[62]
PD-1	Interacting with PD-L1 to put CD8+ T cells into hyporesponsive exhausted state	High expression on CD8+ T cells	[62]
PD-L1	Interacting with PD-1 to put CD8+ T cell into a hyporesponsive exhausted state	High expression on tumor cells	[63]
**Resistance**			
CD4+ Tregs	Immunosuppressive T cells regulating immunity of cytotoxic T cells	High intra-tumoral proportion	[64]
MDSCs	Immune cells secreting immunosuppressive cytokines such as IL-10, IL-35, and TGF-β	High tumor-infiltrated proportion	[65]
M2 macrophages	Anti-inflammatory tumor-associated macrophage secreting immunosuppressive cytokines such as IL-10	High tumor-infiltrated proportion	[61]
LAG-3	Immune checkpoint molecules mediating tumor immune escaping	High expression on LAG-3+ TILs	[66]
TIM-3	Immune checkpoint molecules	High expression on various immune cells	[67]

Factors correlated to response and resistance are separately tabulated under the corresponding sections. Abbreviations: IFN-γ—interferon gamma; TNFα—tumor necrosis factor α; IL-1—interleukin 1; PD-1—programmed cell death protein 1; PD-L1—programmed cell death ligand 1; CTLA-4—cytotoxic T lymphocyte antigen 4; Treg—regulatory T cell; MDSC—myeloid-derived suppressor cell; IL-10—interleukin 10; IL-35—interleukin 35; TGF-β—tumor growth factor β; LAG-3—lymphocyte activation gene 3; TIL—tumor-infiltrated lymphocyte; TIM-3—T cell immunoglobulin and mucin domain-containing protein 3.
